# Bedside Echocardiography for Undifferentiated Hypotension: Diagnosis of a Right Heart Thrombus

**DOI:** 10.5811/westjem.2014.12.23262

**Published:** 2014-12-11

**Authors:** James F. Kenny, Xun Zhong, Cara Brown, Devjani Das, Brock Royall, Monica Kapoor

**Affiliations:** * Northwestern University, Emergency Medicine Residency, Chicago, Illinois; † Staten Island University Hospital, Department of Emergency Medicine, Staten Island, New York

## Abstract

A free-floating right heart thrombus is often a harbinger of a massive pulmonary embolism and must be diagnosed and treated rapidly in order to avoid significant adverse sequelae. We present the case of an 84-year-old female who presented with two days of dyspnea and was hypotensive on arrival. Bedside ultrasound was performed by the emergency physician and showed a large, mobile right heart thrombus leading to immediate administration of a thrombolytic. In this case, bedside ultrasound was utilized to help further delineate clinical care in a progressively worsening patient, leading to a potentially lifesaving treatment.

## INTRODUCTION

A right heart thrombus (RHT) can be differentiated into one of two types: Type A or Type B. Type A thrombus, also referred to as a pulmonary embolus (PE)-in-transit, is often the result of a deep venous thrombosis (DVT). As such, it is worm-like in appearance and freely mobile within the heart chambers.^1^,^2^ In contrast, Type B thrombus is considered to have originated within the atrium or ventricle, and tends to be firmly attached to the chamber wall. Type A thrombus is often more clinically significant as its mobility can markedly impede flow through the right heart, predisposing to rapid cardiovascular collapse and shock.^1^

Given the potentially adverse sequelae associated with right heart thrombi, emergent diagnosis and management are necessary. Multiple studies and case reports have shown that thrombolysis has the potential to rapidly lyse a RHT and decrease mortality while improving right ventricular hemodynamics.^1^,^3^–^7^ In addition, there have been case reports where thrombolytics were not administered, leading to either death or cardiac arrest.^2^,^8^ Thrombolysis, most commonly via tissue-plasminogen activator (t-PA), has the added benefit of rapid administration and availability in institutions where surgery is not feasible.

We present the case of a critically ill patient who continued to deteriorate despite aggressive treatment for presumed sepsis. Bedside ultrasound can be a valuable tool to use during the assessment of a persistently hypotensive patient who is decompensating despite appropriate therapy both in the intensive care unit (ICU) or the emergency department (ED).^1^,^9^ In such patients, the diagnosis of PE associated with RHT may be a rare etiology of refractory hypotension, but should be in the differential diagnosis. We illustrate how the use of bedside ultrasound can diagnose a RHT and alter the management of a critically ill patient in an emergency department setting. We hope this demonstrates that practicing without point-of-care ultrasound can limit an emergency physician’s ability to adequately care for patients with similar presentations.

## CASE REPORT

An 84 year-old female presented to the ED with shortness of breath for the past two days. She was dyspneic on arrival, with noted use of accessory muscles and difficulty answering questions. Her presenting vitals were blood pressure 76/59, respiratory rate 36, oxygen saturation 75% on a non-rebreather mask, a temperature of 100.2°F, and a pulse of 65. Due to her severe respiratory distress and hypoxemia, she underwent endotracheal intubation as she became progressively unresponsive. The working diagnosis was septic shock, so the patient was started on intravenous antibiotics and underwent aggressive fluid resuscitation. A vasopressor, norepinephrine, was empirically initiated.

**Figure 1:**
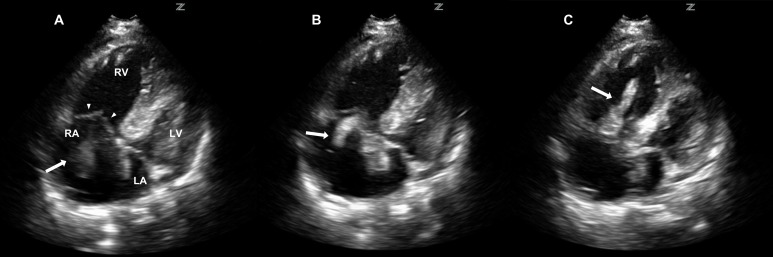
Bedside echocardiography in the four chamber view shows a free-floating atrial clot (arrow) as it glides between the right atrium (RA) and moves to the right ventricle (RV) during cardiac diastole and systole. *A*, the clot (arrow) is seen starting in the RA, *B,* moving through tricuspid valve (arrowheads seen in A), *C,* and finally seen in the RV. *LA*, left atrium. *LV*, left ventricle

**Figure 2:**
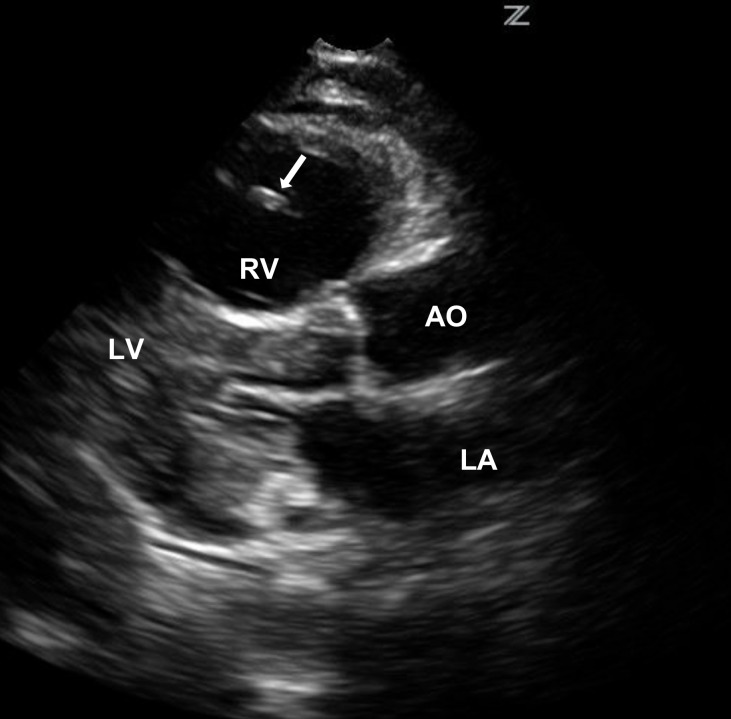
Parasternal long view of the heart with the free-floating clot (arrow) visualized in the dilated right ventricle (RV). *LA*, left atrium; *LV*, left ventricle; *AO*, aortic outflow

Despite the vasopressor, the patient continued to deteriorate. A bedside abdominal ultrasound performed by the emergency physician demonstrated a dilated inferior vena cava (IVC) with minimal respiratory variation. Bedside echocardiography showed no evidence of pericardial effusion, however, a free-floating clot was visualized in the right atrium and ventricle (Figure 1). The right ventricle was dilated and the left ventricle displayed wall hypertrophy and poor contractility (Figure 2 and Video). A presumptive diagnosis of massive pulmonary embolism was made based upon these findings.

Due to the patient’s hemodynamic instability, t-PA was administered after consultation with her family. Subsequent to t-PA administration, the patient’s condition began to improve. She was admitted to the ICU, continued on heparin, and bridged to Coumadin. Her post-t-PA echocardiogram showed complete resolution of the clots, however, right ventricular dilation persisted. The patient continued to improve and was discharged to a skilled nursing facility on hospital day 17.

## DISCUSSION

In a patient with undifferentiated shock, bedside ultrasound can provide vital information for both initial resuscitative efforts and also for identifying alternative diagnoses in patients who are decompensating. In particular, bedside echocardiography can diagnose pericardial effusion, assess global cardiac activity, and visualize abnormalities within the cardiac chambers.^10^,^11^ A free floating RHT is a rare condition that is almost always associated with pulmonary embolism.^12^ Right heart thrombi have been found to occur in 4–18% of cases of acute PE and convey a poorer prognosis as there is a 16–45% mortality rate in these patients.^2^,^13^,^14^ Rapid identification and treatment is therefore essential.

In our case, the patient arrived to the ED hypotensive, hypoxemic, with an elevated temperature, and was immediately treated for sepsis. The patient, however, continued to deteriorate despite aggressive treatment with intravenous fluids and vasopressors. Without bedside echocardiography, the diagnosis would have been delayed, or possibly never found, which could have drastically changed the patient’s outcome.

The utility of bedside focused echocardiography in a patient with hypotension and shock has been embraced by the emergency medicine community, but there remains some concern as to the actual clinical utility of this imaging modality in the emergency department setting.^15^ In our case, the use of bedside echocardiography was valuable in diagnosing a free-floating RHT in an undifferentiated hypotensive patient who was rapidly deteriorating. Without the use of bedside ultrasound, t-PA most likely would not have been administered, and the patient would have continued to decompensate. Focused bedside echocardiography proved to be potentially lifesaving for this patient.

Our goal in presenting this case is to continue to put forth evidence supporting the importance of focused bedside ultrasound, including echocardiography, in the care of a critical patient with a substantial change in clinical condition in an emergency department setting. It is our recommendation that all emergency physicians continue to expand their knowledge of point-of-care ultrasound, particularly in the setting of critically ill and hypotensive patients. This case report illustrates the value of using this tool to diagnose potentially overlooked etiologies of such presentations and should remain an essential component of every emergency physician’s diagnostic repertoire. It is also our recommendation that education regarding the utility of point-of-care ultrasound as a means to drastically alter management and patient care continue to expand in emergency medicine residency programs. While the major limitation of this case report is that it only demonstrates the utility of point-of-care ultrasound in a single patient with a RHT, increasing awareness of RHT as an uncommon, but emergent, disease process has the potential to alter management in critically ill patients in the future.

## Figures and Tables

**Video:** Video of right heart thrombus.
